# Meeting the Educational Needs of Young, ME/CFS Patients: Role of the Treating Physician

**DOI:** 10.3389/fped.2019.00104

**Published:** 2019-04-02

**Authors:** Faith R. Newton

**Affiliations:** Department of Education, Delaware State University, Dover, DE, United States

**Keywords:** myalgic encephalomyelitis/chronic fatigue syndrome (ME/CFS), ME/CFS, chronic fatigue syndrome, educating students with ME/CFS, schools and ME/CFS, education and ME/CFS, classroom accommodations and ME/CFS, academic success and ME/CFS

## Introduction

Myalgic Encephalomyelitis/Chronic Fatigue Syndrome (ME/CFS) is a disabling, chronic disease characterized by the body's inability to produce sufficient energy for normal everyday activities. Children with ME/CFS experience debilitating fatigue referred to as post-exertional malaise (PEM) after minimal mental or physical exertion which is not relieved by sleep. It can significantly reduce the ability of the child to take part in personal, educational, or social activities and can compromise executive function, and can result in a moderate to severe disability. As many as 1% of school-age children suffer from this disease in varying degrees of severity, and ME/CFS has been shown to negatively impact school attendance, participation, connectedness, and academic performance (1). Some studies suggest that ME/CFS may be the major cause of extended school absences (2).

Whereas, the literature supplying practice-based guidance for other chronic conditions affecting children in school, such as Autism and Attention Deficit Hyperactivity Disorder (ADHD) will be found in educational journals, very little guidance for students with ME/CFS appears in the clinical medicine literature. Although school nurses are beginning to play a larger role in supporting these children, physicians or healthcare providers retain primary responsibility of informing the school system of the needed adjustments for the young ME/CFS patient to succeed in the school environment.

This article argues that the physician has a much broader responsibility to provide diagnostic, symptomatic, and treatment information about ME/CFS than they would with other conditions such as Autism or ADHD that qualify students for special services. For students with ME/CFS, the physician's letter required in the school's evaluation process is a critical resource to advise and guide education professionals regarding appropriate student placement, classroom support, and instructional accommodations or modifications. The specifics of what should be included in a model physician's letter are included.

## Paucity of ME/CFS Educational Publications in Comparison to Other Diseases Affecting School Performance

A comparison of available popular and professional literature generally available to educators regarding ME/CFS and the corpus of materials available on Autism and ADHD is instructive. All three conditions impact millions of schoolchildren: ADHD (6.1%), Autism (4.5%), ME/CFS (1%) (3, 4). There are copious practice-based resources available to educators providing services to students suffering from ADHD and Autism. A recent search for popular and professional education resources on Amazon for ADHD returned over 1,000 hits; a similar search for Autism generated 4,000 results. An examination of the titles indicated that, while the quality of the content varies, over 80% of the items were relevant to the topic of educational practices that support students with these conditions.

For ME/CFS, the situation is very different: Even by using multiple terminology references (“CFS” vs. “ME/CFS” vs. “Chronic Fatigue Syndrome,” etc.) and not using specific terms to narrow the results to pediatric ME/CFS, the total number of distinct Amazon returns was only thirty-two in a search performed on October 22, 2018. Of these, 28 proved to be search artifacts; they were non-related items offered by the search engine based on erroneous application of the search terms. Of the four valid results, all were either very general, treated education only as a minor subset of the topic, or included ME/CFS as one of a number of diseases given superficial coverage. Where the easily accessible literature on ADHD and Autism returned hundreds of workbooks, teacher guides, and reference materials for school psychologists, such materials were completely absent for pediatric ME/CFS.

Google searches regarding educational support for the three conditions returned similar results in a search performed on October 22, 2019. While relatively detailed, practitioner-oriented materials for ADHD and Autism are abundant, the best search returns for ME/CFS involve basic checklists about the signs, symptoms, and potential impact of the disease, to which a small number of skeletal bullet points about educational support may or may not be appended.

Conversely, there is a rich, clinical literature regarding ME/CFS in professional medical sources, with a small but significant percentage directly relevant to educational support. A Google Scholar search on October 22, 2018, for peer-reviewed articles, case studies, technical notes, short communications, and reviews on ME/CFS since 2014 currently returns over 18,600 hits; when redundant and erroneous responses are filtered, the number remains high. Nearly 12,000 items (3,000/year) on this disease have been published over the past 4 years, and about 120 (1%) have potential direct applicability to educational support for children suffering from pediatric ME/CFS. The nature of the publications, however, suggests limited availability to education professionals, who are unlikely to be pouring over the pages of *BMC Pediatrics; Brain, Behavior, and Immunity; Current Rheumatology Reports; European Journal of Pediatrics; Frontiers in Pediatrics; Physiotherapy;* or the *Journal of Rehabilitation Medicine*.

## ME/CFS Education-Related Findings in Clinical Medical Literature

Clinicians who remain current in the literature relating to ME/CFS are privy to significant findings that, if known by professional educators, would enhance their ability to enable students suffering from this disease to better achieve academic success. The first general statistical study of school function among students with ME/CFS that extends beyond merely examining attendance and considers the broader impact of the disease on participation, academic performance, and socialization has only recently appeared in *Frontiers in Pediatrics* (1).

Understanding the role of cognitive dysfunction and compromised executive function in school-age children with ME/CFS is essential for teachers attempting to modify lessons and assignments. This knowledge is needed to develop effective instructional strategies that will allow ME/CFS students to succeed. While such resources exist in the clinical medical literature, that information has yet to appear in professional education publications. A summary review of cognitive disfunction caused by ME/CFS appeared in *Current Rheumatology Reports* (5); a comprehensive survey of cognitive/neurological consequences of post-exertional malaise was published in *Brain, Behavior and Immunity* (6), as well as a more directly applicable study of ME/CFS's impacts on cognitive functioning in adolescents in the *European Journal of Pediatrics* (7).

The import of these articles often extends beyond providing teachers a better understanding of the disease and its symptoms. Information contained in these articles can be used directly in assessments for specials services or even in the classroom. *The American Journal of Occupational Therapy* (8) recently published a study that assists teachers in understanding the potential and limitations of students with ME/CFS symptoms. Information contained in these articles, when combined with work illuminating the attention processes of these students in *Behavior, Research and Therapy* (9), comes very close to providing a guide for teachers in mitigating important facets of compromised executive functioning in the classroom. A study in *Physiotherapy* (10) documents the association between ME/CFS pain, comorbidity with other diseases like Fybromyalgia, and cognitive performance. Specific strategies for applying this type of information in schools has appeared in the pages of *Fatigue* (11) and are only beginning to penetrate related literature such as *Journal of School Health* (12).

Journal titles have been mentioned here to make the point that this kind of educationally relevant clinical information has not yet significantly penetrated the mainstream of professional education literature, and is likely to be both unknown and unavailable to school psychologists, guidance counselors, administrators, nurses, and teachers. This situation is beginning to change: Both the Centers for Disease Control and Prevention (13) and the Open Medicine Foundation (14) maintain and update fact sheets on ME/CFS for education professionals, while practice-based articles have begun appearing in publications like *NASN School Nurse* (15). However, there are few professional educators publishing such practice-based material based on current clinical findings, and the bulk of existing material remains relatively inaccessible to educators.

## The Role of the Physician in Supporting Educators

Despite international differences in law and policy, the role of physicians in assisting their school-age patients with disability or disease in gaining access to special services has traditionally centered on documenting for the school the diagnosis, severity, treatment, and prognosis for their patients. Often this occurs through a physician's letter (16). Professional educators then convene in various committees or work groups to address the issue of adapting curriculum, classroom instruction, and other aspects of delivering education based on the student's specific challenges. In most cases, the physician is not an integral part of these groups, though she or he may sometimes be consulted for additional information.

The physician's letter is the most effective document for effecting the needed special services required by students with ME/CFS, because it contains the critical information about the student's condition and limitations. The letter generally remains a permanent part of the student's record during the development of instructional modifications, unlike oral communications or even emails.

## Components of an Effective Physician's Letter Regarding ME/CFS

Providing a diagnosis of ME/CFS is generally not a sufficient guide for educators, because the disease itself is not well-understood, and because ME/CFS is inherently highly variable in severity and symptoms. Physicians need to explain the nature of the disease and the effect of the disease on the individual in question. Here, as in subsequent sections of the letter, references with URL links are exceptionally helpful to educators.

The explanation will normally need to be from 1 to 3 paragraphs in length, and at a minimum cover the following if present in the patient: debilitating fatigue and malaise after minimal exertion; the unpredictability of the severity and length of fatigue; loss of mental/physical stamina with post-exertional malaise; lack of cognitive focus (“brain fog”); orthostatic intolerance; difficulty regulating body temperature; non-refreshing sleep; and myofascial, joint, or abdominal pain. For reference, two of the most useful short summaries of ME/CFS written in language accessible to educators can be found in the Open Medicine Foundation (14) and Centers for Disease Control and Prevention (3) Fact Sheet.

Beyond the general inclusion of diagnosis, severity, treatment, and prognosis, the letter should include extended sections on specific symptoms that will manifest in the classroom, affect school work in general, and recommend changes to the instructional program (either in terms of curriculum, assignments, attendance, or schedule).

With regard to symptoms, more detail is almost always better than less. School officials need to know, for example, that attendance issues and inability to complete a full day's schedule are likely to be chronic issues that, at best, will be slowly responsive to treatment. This notifies and allows them to plan for shortened schedules, consider the deployment of tutors to the homes of chronically absent students, or use educational services over the entire calendar year. Classroom teachers need to be informed that these children will be easily distractible; have difficulty completing sequential tasks or multi-tasking without special support; may demonstrate slower processing speed and difficulty recalling words; and will not be able to “push through” their fatigue to finish assignments. Teachers also need to be aware that one of the cognitive hallmarks of ME/CFS is often the student's inability to self-monitor or self-regulate his or her own fatigue levels.

Detailed recommendations tend to be problematic for physicians, many of whom may be reluctant to impose their views across professional disciplines, while the vocabularies used by the two profession are different. The latter can be an exceptionally high hurdle, as educational terms are often quite different from clinical medical terminology, and have specific procedural and legal connotations. In the United States, for example, the terms “accommodation” and “modification,” when applied to a school setting, are not by any means synonymous, and imply significantly different levels of legal protections for the student and organizational accountability for the school.

However, most educators are open to receiving as much relevant clinical information as possible about a disease they may not have encountered, and about which there is little practice-oriented material. Direct communication, either by email or telephone, with the school psychologist or educational diagnostician who is heading the school's study group for the student will often provide more clarity for the physician regarding what would be helpful in the physician's letter if experience is lacking. The school nurse can provide a critical bridge between the clinician and educators, and is well positioned to become the child's chief medical advocate in the school. Parents can also often provide useful information, although care should be taken to consider the difference between their perspective and that of teachers and school officials.

The most consistently effective method of providing recommendations is to match symptoms directly to recommendations. If symptoms of slow processing speed and limited stamina are present, recommendations of additional time to complete assignments, no penalties for late assignments, and perhaps even a significantly reduced workload provide useful direction. When a patient has demonstrated sensitivity to temperature changes in his or her environment, it is useful to spell out precisely how this should impact the choice of classrooms. If a student needs consistent hydration, there should be an explicit recommendation that any policies against bringing drinks into a classroom should be waived.

It is especially important to address issues of prolonged and unpredictable absences; inability to complete a full school day, or inability to complete homework with specific recommendations. Here the physician should not feel required to go into extreme detail, but to include recommendations about modifying daily schedules, reducing course loads where appropriate, or waiving consequences associated with normal attendance polices (especially the need for multiple doctor's notes for absences). Physicians should also address prognosis; along with unpredictably, educators often do not understand that this disease has a reported recovery rate of 60% by 5 years and by 12 years of ~88% (16).

Finally, it is always useful, as mentioned above, to include references to relevant clinical materials that may assist the school in charting an effective educational course for a student suffering from ME/CFS. Items referenced in this article are a good place to start, but physicians treating pediatric ME/CFS cases should make every effort to stay abreast of new publications that may assist educators.

A sample physician's letter may be found in Rowe (16); while obviously less detailed than that which would be generated in a real clinical case, it follows the general guidelines provided above, and can be used by any physician as a starting point for composition.

## Considerations of Time and Effort on the Part of Physicians

The clinician involvement recommended here is obviously much more laborious than usual for dealing with children suffering from diseases like Autism or ADHD. These demands will also fall more heavily on pediatricians and other clinicians who treat children with ME/CFS, but whose practice is not specifically focused on this disease. As a disease that is diagnosed by exclusion, and with no known cause, ME/CFS is not a psychological illness, however, though depression and anxiety can occur as it does in other chronic illnesses (14).

In terms of patient outcome, the time spent by the physician in assisting the young patient retain educational achievement is time well spent. For patients and their families, dealing with school-related issues consumes far more time and energy on a daily basis than managing medications or any other ME/CFS-abating procedure. From a psychological and motivational standpoint, these families often equate an inability to achieve success in school with long-term negative impacts on their child. Even if the child responds to treatment after months or years, and achieves partial remission of symptoms, the lost semesters at school and the opportunities to complete an education and thereby become a potentially self-sustaining adult, can make dealing with this disease appear daunting. The additional hour expended by the physician in the thoughtful preparation of his or her letter to the school can improve motivation and reduce stress, while simultaneously increasing the chances of successful clinical and educational outcomes for this child.

## Author Contributions

The author confirms being the sole contributor of this work and has approved it for publication.

### Conflict of Interest Statement

The author declares that the research was conducted in the absence of any commercial or financial relationships that could be construed as a potential conflict of interest.
